# Artificial intelligence and machine learning‐aided drug discovery in central nervous system diseases: State‐of‐the‐arts and future directions

**DOI:** 10.1002/med.21764

**Published:** 2020-12-09

**Authors:** Sezen Vatansever, Avner Schlessinger, Daniel Wacker, H. Ümit Kaniskan, Jian Jin, Ming‐Ming Zhou, Bin Zhang

**Affiliations:** ^1^ Department of Genetics and Genomic Sciences Icahn School of Medicine at Mount Sinai New York New York USA; ^2^ Mount Sinai Center for Transformative Disease Modeling Icahn School of Medicine at Mount Sinai New York New York USA; ^3^ Icahn Institute for Data Science and Genomic Technology Icahn School of Medicine at Mount Sinai New York New York USA; ^4^ Department of Pharmacological Sciences Icahn School of Medicine at Mount Sinai New York New York USA; ^5^ Mount Sinai Center for Therapeutics Discovery Icahn School of Medicine at Mount Sinai New York New York USA; ^6^ Department of Neuroscience Icahn School of Medicine at Mount Sinai New York New York USA; ^7^ Department of Oncological Sciences, Tisch Cancer Institute Icahn School of Medicine at Mount Sinai New York New York USA

**Keywords:** Alzheimer's, anesthesia, artificial intelligence, blood‐brain barrier, CNS, depression, disease subtyping, drug design, drug discovery, machine learning, neurological diseases, pain treatment, Parkinson's, schizophrenia, target identification

## Abstract

Neurological disorders significantly outnumber diseases in other therapeutic areas. However, developing drugs for central nervous system (CNS) disorders remains the most challenging area in drug discovery, accompanied with the long timelines and high attrition rates. With the rapid growth of biomedical data enabled by advanced experimental technologies, artificial intelligence (AI) and machine learning (ML) have emerged as an indispensable tool to draw meaningful insights and improve decision making in drug discovery. Thanks to the advancements in AI and ML algorithms, now the AI/ML‐driven solutions have an unprecedented potential to accelerate the process of CNS drug discovery with better success rate. In this review, we comprehensively summarize AI/ML‐powered pharmaceutical discovery efforts and their implementations in the CNS area. After introducing the AI/ML models as well as the conceptualization and data preparation, we outline the applications of AI/ML technologies to several key procedures in drug discovery, including target identification, compound screening, hit/lead generation and optimization, drug response and synergy prediction, de novo drug design, and drug repurposing. We review the current state‐of‐the‐art of AI/ML‐guided CNS drug discovery, focusing on blood–brain barrier permeability prediction and implementation into therapeutic discovery for neurological diseases. Finally, we discuss the major challenges and limitations of current approaches and possible future directions that may provide resolutions to these difficulties.

**Abbreviations**
3D: three‐dimensional
ADME‐T: absorption, distribution, metabolism, and excretion—toxicity
DTI: drug‐target interactions
FDA: the US Food and Drug Administration
HTS: high‐throughput screening
PPI: protein–protein interactions
QSAR: quantitative structure–activity relationship
SAR: structure–activity relationship
SMILES: simplified molecular input‐line entry system

## 1 INTRODUCTION

Disorders of the central nervous system (CNS) are responsible for multiple disease states of significant economic and social impact. Despite huge progress in our understanding of the structure and functions of the CNS, the development of new drugs for CNS disorders poses unique challenges. CNS drugs have lower success rates than other drug classes due to multiple factors, including an insufficient understanding of the pathophysiology of complex CNS conditions, poor target selection/engagement, lack of efficacy in early stages of development, and the presence of a blood–brain barrier (BBB). Such challenges have led to significantly longer development time for CNS drugs, which is, on average, 15–19 years to advance from discovery to regulatory approval.^1^ The whole process of developing a new drug generates a lot of data. Over the past decades, the advances in “omics” technologies, high‐throughput screening (HTS), and chemical synthesis have led to a dramatic increase in the amount of available data on chemical activity^2^ and functional genomics.^3^, ^4^ As a result, how to efficiently combine, correlate, and analyze existing large‐scale data has become a crucial problem for CNS drug discovery.

Artificial intelligence (AI) concepts such as machine learning (ML) have the potential to accelerate pharmaceutical research by extracting novel and important information from the vast amount of complex data generated from the drug discovery process. In recent years, AI/ML‐based methods have been widely applied to many therapeutic areas and achieved state‐of‐the‐art performance in addressing diverse problems in drug discovery. Such applications of AI/ML algorithms also have shown promise in the development of CNS therapeutics—the most challenging area in drug discovery. However, we have only just begun to explore the potential of these technologies for discovering novel therapeutics and repurposing old ones for CNS diseases. Therefore, this review will focus on AI/ML‐assisted drug discovery applications in this promising direction.

Here, we provide an overview of recent developments and applications in AI/ML‐assisted drug discovery, particularly for CNS diseases. This review is intended for biomedical researchers who are curious about the potential of AI/ML for advancing CNS drug discovery and consider AI‐based tools in their research. We first provide a broad overview of AI/ML approaches in drug discovery and then review AI/ML solutions to the issues in drug discovery specific for CNS diseases. We start with a brief introduction to AI algorithms and their input molecular descriptors and then summarize AI/ML‐based methods in various stages of drug discovery, including target identification and characterization, virtual screening, lead discovery, and physicochemical pharmacokinetic property prediction. We further review recent AI/ML applications in *de novo* design, predicting drug sensitivity and response, drug synergy prediction, and drug repurposing. For CNS diseases specific drug discovery, we focus on AI/ML solutions to key challenges such as BBB permeability and introduce AI/ML‐assisted applications to neurological diseases, including neurodevelopmental disorders, depression, Parkinson's disease, Alzheimer's disease, anesthesia and pain treatment. We conclude the review by highlighting challenges, limitations, and future directions of AI/ML‐aided drug discovery, especially for CNS diseases.

## 2 AI/ML APPLICATIONS IN DRUG DISCOVERY

**Figure 1:**
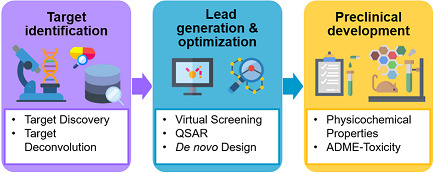
AI/ML applications in the drug discovery pipeline. AI/ML approaches provide a range of tools that can be applied in all the three stages of early drug discovery to improve decision making and speed up the process. ADME, absorption, distribution, metabolism, and excretion; AI, artificial intelligence; ML, machine learning; QSAR, quantitative structure–activity relationship

AI/ML has been utilized at three different stages of early drug discovery process, including target identification, lead generation and optimization, and preclinical development (Figure 1). In target discovery, AI‐based approaches have been used to integrate heterogeneous data sets to identify patterns so as to understand molecular mechanisms underlying diseases and drug activities. For lead generation and optimization, AI/ML algorithms improve the scoring functions and quantitative structure–activity relationship (QSAR) models in virtual screening pipelines and support the automation and optimization of the de novo drug design processes. In preclinical development, AI/ML approaches are employed to generate predictive models of physicochemical properties by efficiently processing large amount of chemical data and further optimize absorption, distribution, metabolism, and excretion—toxicity (ADME‐T) profiles.

### 2.1 Overview of the AI/ML algorithms

**Figure 2:**
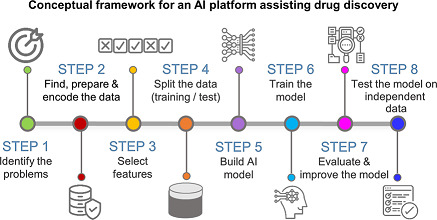
The basic steps of building an artificial intelligence (AI) platform for drug discovery. The process for developing an AI model as follows: (1) Define the problem appropriately (objective, desired outputs, etc.), (2) prepare the data (collection, exploration and profiling, formatting, and improving the quality), (3) transform raw data into features and select meaningful features (a.k.a. feature engineering), (4) split data into training and validation sets, (5) develop a model, (6) train the model with a fraction of the data, test its performance (cross‐validation) and tune its parameters with the validation set (7) evaluate model performance on the validation set and refine the model, and (8) evaluate the model on independent data not used for method development

To help the reader better understand AI/ML applications in CNS drug discovery, we provide a summary of AI‐based algorithms that are widely used in drug discovery. AI uses a large variety of models to build up intelligent systems, which can be classified by learning procedures. AI is frequently used to denote ML algorithms—yet they are not the same. So, it would be worth clarifying both terms at first. In this review, we follow the US Food and Drug Administration's (FDA) definition of AI. They describe AI as “the science and engineering of making intelligent machines”, while ML is “an artificial intelligence technique that can be used to design and train software algorithms to learn from and act on data”,^5^ adding that all ML techniques are AI techniques, but not all AI techniques are ML techniques. Here, we provide brief definitions of the basic learning algorithms in Table 1, as these are most relevant in the context of drug discovery. AI‐related learning techniques are broadly categorized as supervised, unsupervised, semisupervised, active, reinforcement, transfer, and multitask learning. Different algorithms are used in those learning architectures to perform specific tasks such as classification or clustering. However, success with AI requires more than training an AI model. A robust AI workflow involves (i) formulating a problem, (ii) preparing data, (iii) extracting features, (iv) selecting training and testing data sets, (v) developing a model, (vi) training the model and testing its performance (cross‐validation), and (vii) applying the model to testing data sets and refining the model. Figure 2 displays the basics steps of building an AI architecture.

**Table 1:** AI‐related learning techniques used in drug discovery

Category of learning	Definition	
**Supervised learning**	A predictive model trained on data points with known outcomes (“labeled data”) Two types of problems:	
	**Regression**: Model finds outputs that are real variables	
	**Classification**: The model divides inputs into classes or groups	
**Algorithm**	**Task**	**Description**
Naïve Bayes	Classification	A “probabilistic classifier” that determines the probability of the features occurring in each class by treating every feature independently to return the most likely class based on the Bayes rule. Particularly suited when the dimensionality of the inputs is high.
Support vector machines	Classification	A discriminative classifier that outputs an optimal hyperplane to categorize new examples. The vectors that define the hyperplane are the support vectors.
Random Forest	Classification/Regression	An ensemble of simple tree predictors that vote for the most popular class for classification problems. In the regression problems, the tree responses are averaged to obtain an estimate of the dependent variable. Overfitting is less likely to occur as more decision trees are added to the forest.
K‐nearest‐neighbors	Classification/Regression	A nonparametric algorithm based on feature similarity by assuming that similar things exist in close proximity. Useful for a classification study when there is little or no prior knowledge about the distribution data.
Artificial neural networks	Classification/Regression	A method that learns from input data based on layers of connected neurons consisting of input layers, hidden layers, and output layers.
Deep neural network	Classification/Regression	A collection of neurons organized in a sequence of multiple layers. Type of artificial neural network with several advantages (i.e., shared weights [parameter sharing), spatial relations, and local receptive fields Learning can be supervised, unsupervised, or semisupervised. End‐to‐end learning and transfer learning are the major approaches performed by the deep neural network. Autoencoders and generative adversarial networks are the two specific forms of deep neural networks.
Multiple regression	Regression	A statistical approach to find relationships between dependent variables and one or more independent variables.
**Unsupervised learning**	A self‐organized model that organizes the data in some way or describe its structure to learn underlying patterns of features directly from unlabeled data.	
**Algorithm**	**Task**	**Description**
K‐means clustering	Clustering	A classification method that divides data into k groups by minimizing within‐group distances to the centroid
Fuzzy clustering	Clustering	A form of clustering (Fuzzy C‐means clustering) in which each data point can belong to more than one cluster. It computes the coefficients of being in the clusters for each data point.
Hierarchical clustering	Clustering	A classification method that builds a hierarchy of clusters by merging two close clusters into the same cluster. This algorithm ends when there is only one cluster left.
Principal component analysis	Dimensionality reduction	A nonparametric statistical technique that uses an orthogonal procedure to transform a set of correlated features to new independent variables called principal components
Independent component analysis	Dimensionality reduction	A statistical method that separates a multivariable output into statistical independent additive components
Autoencoders	Dimensionality reduction	A deep neural network trained with backpropagation to reconstruct its original input
Deep belief nets	Dimensionality reduction	Probabilistic generative models with many layers of stochastic, latent variables. Each layer is a Restricted Boltzmann machine.
Generative adversarial networks	Anomaly detection	Deep generative models that use two neural networks, pitting one against the other (thus the “adversarial”) to generate new synthetic but realistic instances of data.
Self‐organizing map	Dimensionality reduction	A competitive learning network that reduces the input dimensionality to represent its distribution as a map.
**Semisupervised learning**	A combination of supervised and unsupervised learning methods that uses a small amount of labeled data and also a large amount of unlabeled data during training to gain more understanding of the sample population.	
**Active learning**	A particular case of semisupervised learning, where the algorithm is allowed to query the user for the label of a subset of training instances Used to construct a high‐performance classifier while keeping the size of the training data set to a minimum by actively selecting the valuable data points	
**Reinforcement learning**	Dynamic programming that trains algorithms using a system of reward and punishment to maximize the performance.	
**Transfer learning**	A deep learning technique enables developers to harness a neural network used for one task and apply it to another domain. It allows the reuse of a pretrained deep neural network on a new task with only a small amount of data. Useful when the data is insufficient for a new domain to be handled by a neural network, and there is a big preexisting data pool that can be transferred	
**Multitask learning**	An approach to inductive transfer that improves generalization performance of multiple related tasks by leveraging useful information among them. Useful when there are multiple related tasks, each of which has limited training samples	
**Multiple kernel learning**	A flexible learning method that use a predefined set of kernels and learn convex combinations of kernels over potentially different domains. Used when there are heterogeneous sources of data for the task at hand	
**Ensemble learning**	A meta‐algorithm that combines decisions from multiple models into one predictive model to decrease variance (bagging), bias (boosting), or improve predictions (stacking).	
**End‐to‐end learning**	A deep learning process in which all of the parameters are trained jointly, rather than step by step. It allows the training of a deep neural network based on raw data without descriptors. Since the pipeline is replaced with a single learning algorithm, it goes directly from the input to the desired output and thereby overcome limitations of the traditional approach.	

*Note*: The rows with gray backgrounds show the basic learning categories and their definition, while the rows following supervised and unsupervised learning parts display the different algorithms used in these categories.

### 2.2 Molecular descriptors and fingerprints for input data preparation

A key consideration in early drug discovery is to identify drug candidates with the desirable initial characteristics, which are then further developed into chemical structures with the desirable potency against the target molecule. Molecular descriptors and fingerprints are used for quantifying such physicochemical characteristics of both chemical entities and their biological target molecules. Molecular descriptors are experimentally quantified or theoretically characterized properties of a corresponding molecule that represent the physical, chemical, or topological characteristics, while molecular fingerprints are more complex descriptors that are encoded as binary bit strings.^6^, ^7^ Both molecular descriptors and fingerprints have crucial functions in ML‐based applications in drug discovery processes such as target molecule ranking,^8^, ^9^ similarity‐based compound search,^10^, ^11^, ^12^, ^13^, ^14^, ^15^ virtual screening,^16^, ^17^ QSAR analysis,^18^, ^19^ ADME‐T prediction of lead molecules.^20^, ^21^, ^22^, ^23^

There are various tools for molecular descriptor and fingerprint calculation, and each has a different set of features. Here, we explain the molecular descriptors (i.e., target protein descriptors and compound descriptors) and compound fingerprints, and provide the highly used programs for generating them (i.e., sequence‐based tools and structure‐based tools) in the Supporting Information. Additionally, Chuang et al.^24^ comprehensively discussed how AI‐based methods (i.e., deep learning [DL]) could address limitations of molecular descriptors and fingerprints and thereby improve the predictive modeling of compound bioactivities.

### 2.3 AI/ML applications in target identification

A dominant approach to drug discovery is to design drug molecules that will reverse a disease course by modulating the activity of a target.^25^ Drug development often begins with identification of a novel target whose modulation can lead to a therapeutic benefit with an acceptable safety margin. This is followed by validating the role of the selected target in disease in in vivo models and, ultimately, in clinical trials. Therefore, the ultimate success of a drug development project depends on early identification of promising drug targets.

A good drug target need be relevant to the disease phenotype as well as be suitable for therapeutic modulation (“druggable”). Biological and technological advances have continuously driven the generation of high‐throughput biomedical data, which present new opportunities for early identification of potential drug targets. However, the analysis of such large‐scale multidimensional biological data requires effective techniques that can produce accurate predictions for target identification. AI/ML has emerged as a powerful technology for analyzing the rapidly increasing multiomics data in the identification of potential therapeutic targets.

In literature, the “target identification” term is often used in two different contexts: Target discovery and target deconvolution.^26^ The first is the discovery of a new disease target whose modulation would have therapeutic effects. The second is the identification of a target with a known active compound, which is also called “target fishing.” To avoid confusion, we will use context‐specific terms of target discovery and deconvolution rather than generic target identification.

#### 2.3.1 Target discovery

**Figure 3:**
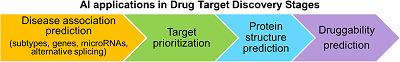
AI‐guided target discovery. AI/ML methods can efficiently analyze all available information to speed up the discovery of disease‐related drug targets. Specifically, AI/ML methods are utilized for disease subtyping, identification of disease driver genes and microRNAs, alternative splicing prediction, triaging of novel drug targets, modeling of three‐dimensional target structures, and druggability assessment. AI, artificial intelligence; ML, machine learning

Drug discovery begins with the identification of a novel target candidate that is followed by a target evaluation consisting of experimental target validation and theoretical assessment of its ability to bind small molecule drugs (druggability).^27^ The target discovery process includes identification of targets that play a role in the disease pathophysiology,^28^ assessment of druggability, and prioritization of candidate targets. However, because of the complex nature of human diseases, this process often requires more comprehensive approaches that integrate available heterogeneous data and information to understand the molecular mechanisms underlying disease phenotypes and identifying the patient‐specific changes.^29^ To overcome such difficulties, researchers have applied AI/ML methods to predict “reliable” drug targets. The following sections demonstrate the AI/ML applications in different stages of the target discovery process (Figure 3).

##### 2.3.1.1 Disease subtype prediction

In complex heterogeneous diseases, classifying patients into clinically and biologically homogenous subtypes is critical for understanding disease pathophysiology and developing appropriate subtype specific therapies.^30^ Researchers have developed AI/ML algorithms that can integrate multiscale data to identify different etiological subtypes of complex diseases. For example, Shen et al.^31^ developed iCluster, a joint latent variable model for integrative clustering analysis, which was applied to breast cancer and lung cancer and identified subtypes characterized by concordant DNA copy number changes and gene expression.^31^ Yuan et al.^32^ also integrated copy number variation and gene expression data by using a nonparametric Bayesian model and discovered prognostic subtypes in prostate cancer and breast cancer.^32^ Zhang et al.^33^ revealed the prognostic subtypes in neuroblastoma using DL‐based integration of multi‐Omics data and K‐means clustering analysis. Recently, Gao et al.^34^ described a cancer classification method, deep cancer subtype classification (DeepCC), based on DL of functional spectra, which is a vector of gene set enrichment scores associating with biological functions for each patient sample. Overall, in recent years, AI/ML methods have been employed to analyze large‐scale genomic and other molecular profiling data in cancer for the identification of distinct, molecular disease subtypes. However, such AI‐based subtyping analysis have not been widely applied to other complex diseases. Implementation of robust and scalable AI/ML techniques for discovery of disease subtypes paves the way for developing more efficacious therapeutic strategies.

##### 2.3.1.2 Prediction of disease driver genes

One of the most challenging tasks in target discovery is the prediction of disease‐causing genes from huge amount of genetic and functional genomic data. To predict these disease‐associated genes from multiomics data, researchers have employed various ML classifiers,^35^, ^36^, ^37^, ^38^ including Random Forest (RF)‐,^39^, ^40^ support vector machines (SVM)‐,^41^, ^42^ and decision tree (DT)‐based classifiers.^43^ More detailed information about those applications can be found in the Supporting Information. Besides the ML‐methods using multiomics data, DriverML,^44^ a supervised learning tool, identified cancer driver genes based on DNA sequence alterations from The cancer Genome Atlas (TCGA) data with superior performance over the other tools such as DriverDBv2 database.^45^

In addition to ML classifiers, DL‐based methods have been implemented in more recently developed tools. For example, deepDriver^46^ trained similarity networks and a convolutional neural network (CNN) on mutation data simultaneously to predict driver genes with better performance than the competing approaches when applied in breast cancer and colorectal cancer. In another example, Peng et al.^47^ used deep neural network (DNN) to reduce the dimensionality of transcriptomics data to predict Parkinson's disease genes. This DNN‐based tool, namely, N2A‐SVM, consists of three steps, including extraction of vector representation of each gene in the protein–protein interaction (PPI) network, dimension reduction for the obtained vector with autoencoder, and prediction of the genes associated with Parkinson's disease using SVM.

Multitask learning has also been employed for the prediction of cancer driver genes. LOTUS, an ML‐based algorithm, predicts cancer driver genes in a pan‐cancer setting, as well as for specific cancer types, using a multitask learning strategy sharing information across cancer types.^48^ For the readers who want to learn more about opportunities and challenges in predictive modeling for multiomics data sets, we suggest the review paper of Kim and Tagkopoulos.^49^

Different from the tools using omics data sets, BeFree^50^ was developed to extract relations between genes and diseases from text mining. This supervised learning approach utilized natural language processing (NLP) Kernel methods to identify gene–disease associations from the abstracts collected by Medline.

##### 2.3.1.3 Prediction of disease‐associated microRNAs

The challenges in targeting disease proteins have shifted the focus in target selection to disease microRNAs (miRNAs), which are small noncoding RNAs that regulate gene expression by targeting messenger RNAs.^51^ miRNAs are regarded as high‐potential drug targets due to their involvement in various diseases.^52^ Therefore, considerable effort has been devoted in identifying relationships between miRNAs and diseases using ML‐based methods, such as the network based approach by Xu et al.^53^, ^54^ and RLSMDA. New strategies in miRNA target discovery have utilized neural networks (NN). Zeng et al.^55^ developed a NN method, NNMDA to predict miRNA‐disease associations with the best performance among the existing algorithms. Application of NNMDA to lung neoplasm and breast neoplasm predicted novel disease‐related miRNAs. Very soon after that, Zheng et al.^56^ published a new ML‐based method, MLMDA, which predicts miRNA–disease associations by integrating miRNA sequence, disease semantics, miRNA–disease association, and miRNA function but with slightly worse performance than NNMDA.

##### 2.3.1.4 Prediction of alternative splicing

Alternative splicing (AS) plays a fundamental role in gene expression regulation and protein diversity by causing the generation of different transcripts from single genes.^57^ Understanding the genetic variation in splicing signals is within the scope for AI/ML‐based models to discover therapeutic opportunities through novel targets. For splicing prediction and analysis, a web tool, AVISPA,^58^ has been developed. For a given exon and its proximal sequence, AVISPA predicts if the exon is alternatively spliced and if it has associated regulatory elements by using a Bayesian NN classifier. However, the method by Leung et al.^59^ outperformed the Bayesian NN approach for predicting AS by developing a DNN model inferred from mouse RNA‐Seq data that can predict splicing patterns in individual tissues and differences in splicing patterns across tissues. Later, Jha et al.^60^ compared those two previous modeling approaches, Bayesian and Deep NN, and determined the confounding effects of data sets and target functions. On the basis of this knowledge, they developed a new target function for AS prediction with higher accuracy. For further improvement of the prediction, they developed a modeling framework that uses transfer learning to combine CLIP‐Seq, knockdown, and overexpression experiments. For enabling the usage of unlabeled data and the latent information, Stanescu et al.^61^ applied semisupervised learning algorithms to AS prediction. Xiong et al.^62^ built up a DL model trained to predict splicing from DNA sequence alone and successfully identified new autism‐linked genes.

##### 2.3.1.5 Target prioritization

While increasing effort has been devoted to nominating novel drug targets involved in diseases, experimental validation of identified target candidates is an expensive and time‐consuming task.^63^ Therefore, researchers have utilized AI/ML approaches to support the prioritization of the most promising target candidates for subsequent experiments. To identify and prioritize novel cancer drug targets, Jeon et al.^64^ built an SVM classifier that uses features from various data types (DNA copy number, messenger RNA expression, mutation occurrence, and PPI) to prioritize drug targets specific for breast, pancreatic and ovarian cancers. To improve the disease gene prioritization process, Valentini et al.^65^ combined different functional gene networks and applied a kernel‐based method to prioritize genes according to the disease MeSH terms. Then, Ferrero et al.^66^ took advantage of the publicly available target–disease association data from the open targets platform training an NN classifier with semisupervised learning and predicted novel therapeutic targets. As another publicly available data source, Medline abstracts also have been benefited for developing prediction tools (i.e., DigSee^67^) that identify disease–gene relationships and prioritize the genes based on evidence. Specifically, DigSee uses NLP to extract the relationship between diseases and genes and ranks the evidence sentences with a Bayesian classifier. Recently, Arabfard et al.^68^ predicted and prioritized over 3,000 candidate age‐related human genes using three positive unlabeled learning algorithms, Naïve Bayes, Spy, and Rocchio‐SVM. They ranked the human genes according to their implication in aging based on binary gene features from 11 human biology databases.^68^

##### 2.3.1.6 Target protein structure prediction

AI/ML architectures have been applied in protein structure prediction over 30 years, and several groups have comprehensively reviewed those strategies.^69^, ^70^, ^71^, ^72^, ^73^ Therefore, we will focus on recent applications in this field. Also, we provide a background of conventional protein structure prediction methods (i.e., template‐based and template‐free) for those who want to learn more about this field in the Supporting Information.

Since 1994, the Critical Assessment of protein Structure Prediction (CASP) competitions have been organized biannually for blind evaluation of the state‐of‐the‐art methods that predict three‐dimensional (3D) protein structures from protein sequences. There, each group submits structure predictions for each of the given protein sequences for which experimentally determined structures were sequestered. In December 2018, Google's AI firm DeepMind won the CASP13 competition with its latest AI system, AlphaFold. DeepMind's success generated significant interest in the protein folding community, where the researchers published several articles discussing the method.^74^, ^75^, ^76^, ^77^ AlphaFold determines the 3D shape of a protein from its amino acid sequence by merging two approaches: (i) Inferring physical contact in protein structure from residue covariation in protein sequence based on coevolution analysis of a multiple sequence alignment and (ii) identifying coevolutionary patterns in protein sequences as contact distributions by using DNNs and convert them into protein‐specific statistical energy potentials. AlphaFold system has achieved an unprecedented prediction accuracy among the ab initio methods. Although AlphaFold's performance represents a big leap in protein structure prediction, its accuracy still needs to be improved.

Inspired by AlphaFold as well as previous successful applications of DL to residue contact predictions,^78^ researchers have developed different strategies to improve the protein structure prediction, including a deep residual network model,^79^ a fragment library that is built using deep contextual learning techniques called DeepFragLib^80^ and a community‐built, open‐source implementation of Alphafold (i.e., ProSPr).^81^ The emergence of DL has suggested the rethinking of how to address the problem of protein structure and thereby, encourages the new approaches. RGN (recurrent geometric network) is an end‐to‐end differentiable model that takes a sequence of amino acids and position‐specific scoring matrices (a summary of residue propensities for mutation) as inputs and outputs a 3D structure. In contrast to the complexity of conventional structure prediction models, a trained RGN model is a single mathematical function that is evaluated once per prediction. Hence, a trained RGN makes predictions six to seven orders of magnitude faster than other methods. The same lab developed the RGN also published a data set to provide a standardized resource for training and assessing ML frameworks for predicting protein structures. The data set called ProteinNet integrates sequence, structure, and evolutionary information into preformatted input/output records. ProteinNet is available in a public repository, https://github.com/aqlaboratory/proteinnet.

Going beyond the structure prediction, researchers have employed the ML for the prediction of protein dynamics since target proteins are dynamic and sample multiple states. Ung et al.^82^ used RF to classify pharmacologically relevant conformations of protein kinases. Using a 3D‐CNN, Okuno et al.^83^ developed DEFMap, which extracts the dynamics information hidden in a given cryo‐EM density map. This approach allows us to grasp the dynamic changes associated with molecular recognition and the accompanying conformational selections from the cryo‐EM structure, which derive insights into the protein function as well.

The studies discussed above clearly demonstrate the utility of the AI/ML frameworks to make predictions of protein structural features from sequence alone. Rost et al.^84^ comprehensively discussed how ML algorithms help to understand the effects of protein sequence variants on protein function and pathways. AI/ML algorithms are readily available for structural biologists to quickly estimate protein structures. Of course, the accuracy and speed of a framework will depend on the creativity in problem formulation, network design, and data storage. We can look forward to a rapid growth in the number of AI/ML applications in the prediction of protein structures.

##### 2.3.1.7 Druggability

In target discovery, another crucial step is the evaluation of the target's druggability, “the likelihood of being able to modulate a target with a small‐molecule drug”.^85^ In drug design, a selected target must have the biophysical properties that allow it to bind small molecules with drug‐like properties. ML‐based models usually estimate a target's druggability by using different features of it. As one of the earliest applications, SCREEN (Surface Cavity REcognition and EvaluatioN) webserver^86^ was built based on an RF classifier trained on geometric, structural, and physicochemical features of drug‐binding and nondrug‐binding cavities on proteins. The classification process reveals that the most critical attributes to estimate druggability are the size and shape of the surface cavities of the protein. In the following studies, SVMs were applied to predict druggable targets based on various physicochemical properties from protein sequences.^87^, ^88^ Then, Costa et al.^89^ constructed a DT‐based meta‐classifier by training on attributes including network topological features, tissue expression profile, and subcellular localization for each druggable and nondruggable gene. Later, Wang et al.^90^ combined a biased SVM with a DL model, stacked autoencoders, to identify drug target proteins based on the sequence information of proteins. Recently, Kokh et al.^91^ developed an ML tool for the druggability analysis of binding pocket variations during the protein movement. They used a logistic regression model and a CNN to identify potentially druggable protein conformations in trajectories from molecular dynamics simulations. On the contrary, Dezső and Ceccarelli^92^ built up RF models for the druggability prediction of oncology drug targets to prioritize proteins according to their similarity to approved drug targets. More details on ML‐based tools designed to predict the druggability of targets can be found in the review from Kandoi et al.^93^

#### 2.3.2 Target deconvolution

Target deconvolution (a.k.a. target fishing) is an important step following the discovery of compounds that cause a desirable change in phenotype. Understanding the binding targets of phenotypic screen‐derived compounds can help design better analogs, find potential off‐targets, and thereby explain observed adverse events. However, existing experimental approaches for target deconvolution are labor, resource, and time‐intensive. Researchers have adapted computational approaches to target deconvolution problems to reduce the required sources for the experiments. Several studies implemented AI/ML algorithms into computational target deconvolution tools for higher predictive power. For example, Schneider and colleagues have widely applied self‐organizing maps (SOMs) to predict the macromolecular targets of compounds.^94^, ^95^, ^96^, ^97^ They preferred to use “fuzzy” molecular representations, such as pharmacophoric feature descriptors, since such fuzzy molecular representations demonstrated greater scaffold‐hopping potential than atomistic approaches in similarity searches. On the basis of the similarity of pharmacophoric features, their unsupervised SOM algorithm clustered the query molecules with unknown targets as well as drug‐like molecules with known targets. Hence, the trained SOM was able to transfer the knowledge of annotated drug targets to query molecules that are the nearest neighbors to known drugs.^94^ They have applied this SOM approach to identify the macromolecular targets of de novo‐designed molecules,^95^ complex natural products,^94^ fragment‐like natural products,^96^ and a natural anticancer compound.^97^ Besides the SOM models, a multiple‐category Naïve Bayesian model was developed for the rapid identification of potential targets for compounds based on only chemical structure information, which is the connectivity fingerprints of compounds from 964 target classes in the WOMBAT (World Of Molecular BioAcTivity) chemogenomics database.^98^ Moreover, a target‐fishing server named RF‐QSAR was built based on target SAR models that were created using an RF algorithm to rank candidate targets for a query compound.^99^ A recent target identification tool, BANDIT,^100^ uses a Bayesian approach to integrates six distinct data types—drug efficacies, posttreatment transcriptional responses, chemical structures, reported side effects, bioassay results, and known targets.

In the identification of the novel targets of drugs, there has been increasing interest in predicting drug–target interaction (DTI), given its relevance for side effect prediction and drug‐repositioning attempts.^101^ The availability of heterogeneous biological data on known DTI has enabled the development of various AI/ML‐based strategies to exploit unknown DTI,^102^ including ensemble learning,^103^, ^104^, ^105^, ^106^ tree‐ensemble learning,^107^ active learning,^108^ DL,^109^ end‐to‐end DL,^110^ and kernel‐based learning.^111^, ^112^, ^113^, ^114^, ^115^ Such AI/ML‐enabled data integration strategies outperform the traditional methods in classifying both positive and negative interactions,^110^ improved the quality of the predicted interactions, and expedited the identification of new DTI.^115^

### 2.4 AI/ML applications in compound screening and lead discovery

To identify new compounds with potential interactions to target proteins, researchers commonly use HTS, an in vitro method that automatically tests large compound libraries towards a specific target. However, high cost and low hit rate of HTS have expedited the development of *virtual screening* (VS) alternatives, which enable cheaper and faster screening of larger compound libraries.^116^, ^117^ VS predicts the compounds that most likely to bind to a protein of interest using various approaches. Two broad categories of VS are structure‐based VS (SBVS) and ligand‐based VS (LBVS)—the former takes the structures of target proteins as input,^118^, ^119^ and the latter uses information on known inhibitors.^120^ LBVS is basically “analoging” to some extent based on that similar molecules tend to exhibit similar properties,^121^ and it also helps to build better pharmacophore models. SBVS and LBVS are often used synergistically: Leads from SBVS can be improved with LBVS, and data from improved yields can be used to refine models for SBVS.^122^ For achieving better performance in VS workflows, AI/ML‐based methods have been utilized for both SBVS and LBVS. We will begin with the application of AI/ML methods in SBVS and continue with their applications in LBVS in the next section.

#### 2.4.1 Structure‐based virtual screening

SBVS requires the 3D structure of a target protein to predict whether a compound is likely to bind the target. One widely used method to do this is molecular docking, which models the protein–ligand complex based on the estimated interaction energy. In recent years, ML methods have been employed in SBVS workflow to increase the robustness and accuracy of scoring functions (SFs), conformational sampling and ranking. Researchers have developed SFs using RF‐,^123^, ^124^, ^125^, ^126^ SVM‐,^127^, ^128^ and NN‐^129^, ^130^, ^131^, ^132^, ^133^, ^134^ based learning algorithms and they outperformed the conventional SF predictions.^135^ However, no ML‐based SF is superior to all the other approaches in all respects.^136^ Indeed, the performance of an SF differs from target to target.^137^ Therefore, researchers have developed ML‐based, target‐specific SFs to improve the efficiency of existing SFs for kinases,^138^, ^139^, ^140^, ^141^ histone methyltransferases,^142^ cyclin‐dependent kinases and G protein‐coupled receptors (GPCRs),^137^ and cytochrome P450 aromatase.^143^

Moreover, such ML‐based models have been applied to post‐docking processes to improve the accuracy of molecular docking. For example, ML algorithms^142^, ^144^, ^145^, ^146^, ^147^, ^148^ improve pose/compound selection by automating the evaluation of docked ligands, which was done manually before.^149^ Details about ML‐based scoring functions and AI/ML applications in the post‐docking stage can be found in the Supporting Information.

#### 2.4.2 Ligand‐based virtual screening

When the 3D structure of a given target is available, SBVS approaches (i.e., molecular docking) can be employed. However, LBVS methods are the only option if the 3D structure of the target protein is not known. In contrast to the molecular docking that predicts the binding pose of ligands to the target protein using the protein structure, LBVS is based on the principle that ligands structurally similar to an active compound tend to have similar activity.^150^ Hence, LBVS requires the information of known active compounds rather than the target protein structure. In drug discovery efforts, researchers often have a set of active compounds generated from testing molecules in biochemical or functional assays without knowing the target protein structure. In such cases, the LBVS approach can be utilized to find new ligands by assessing the structural similarity of candidate ligands to the known active compounds. The challenge is thereby to find an appropriate model for similarity that relates compound features to assay outcomes. In recent years, ML has emerged as an attractive approach to boost the predictive power of LBVS models. The specific aims of ML approaches include prediction of the active compounds against a particular target using models trained on input data sets, discrimination of drug modules from nondrug ones, and prioritization of compounds based on the probability of activity. For these purposes, researchers have used SVMs, Bayesian architectures, and artificial neural networks (ANNs) (Table S2). Further information regarding AI/ML applications in LBVS is available in some comprehensive review papers.^136^, ^151^, ^152^

On the contrary, one of the most recent advances in AI/ML‐based LBSV was made by Stokes et al.^153^ They successfully discovered new antibiotics by employing graph convolutional networks (GCN), whose outstanding performance over conventional ML models in predicting molecular properties was confirmed by two studies.^154^, ^155^ Using their GCN model, the authors performed a large‐scale screening and identified a promising new antibiotic, halicin.^153^

In conclusion, the advances in selection and design of AI/ML algorithms for LBVS and the availability of large bioactivity data sets have enabled more accurate and faster selection of compounds that are predicted to be active against a particular target and will undergo further experimental assays eventually. Although traditional ML classifiers had been widely used in LBVS, recent successful applications have shown GCN's potential to become a popular approach for LBVS.^151^

#### 2.4.3 QSAR prediction

QSAR models are developed to identify a mathematical relationship between the physicochemical properties, which are represented by molecular descriptors, and biological activity of chemicals. These models play a prominent role in drug optimization, providing a preliminary in silico evaluation of essential attributes related to the activity, selectivity, and toxicity of candidate compounds.^156^, ^157^, ^158^ By doing that, they significantly reduce the number of candidate compounds to be tested by in vivo experiments. QSAR models can be based on regression or classification models that depend on the underlying computational strategy. AI/ML approaches (i.e., RF,^159^, ^160^ SVM,^161^, ^162^, ^163^ Naïve Bayesian,^164^, ^165^, ^166^, ^167^, ^168^, ^169^, ^170^, ^171^, ^172^, ^173^ and ANN^143^, ^174^, ^175^, ^176^, ^177^, ^178^, ^179^, ^180^, ^181^, ^182^, ^183^, ^184^) have been extensively employed in QSAR modeling (For the detailed discussion of the applications, see the Supporting Information). Notably, the RF algorithm is commonly used as a classification and regression tool^159^ and considered to be the golden standard in QSAR studies.^185^ Hence, the performance of new QSAR prediction tools often is compared with that of RF. Many RF‐based QSAR models have been developed, such as pQSAR,^186^ a method for the soluble epoxide hydrolase,^187^ and a model for Janus kinase 2.^188^ When the predictive performance and interpretability of RF‐based QSAR models are compared to those of two widely used linear modeling approaches—SVMs and partial least‐squares, RF not only yields better predictive performance but also enables an amenable chemical and biological interpretation.^189^

In the applications of NN to QSAR prediction, researchers use the data from a single assay using molecular descriptors as input to train an NN and record activities as training labels. However, the efficiency of those simple single‐task NN models depends on having sufficient training data in a single assay. To benefit from the data obtained from multiple assays, researchers aim to develop multitask QSAR models. Several groups constructed the multitask learning structures based on plain feed‐forward NN to avoid overfitting by learning multiple bioassays simultaneously.^190^, ^191^, ^192^, ^193^, ^194^, ^195^, ^196^ Moreover, multitask QSAR models were also utilized for predicting the activity against multiple targets.^197^, ^198^, ^199^

In 2012, a data science competition (www.kaggle.com/c/MerckActivity) was organized to find state‐of‐the‐art methods for QSAR. Using multitask DNNs, the winning team improved the prediction accuracy by 15% over the baseline RF method.^200^ Since its introduction into the QSAR modeling,^159^ RF has served as a “golden standard” and no QSAR methods other than DNNs outperform it. On the contrary, in the following DREAM challenges on predicting kinase‐drug‐binding,^201^ the models based on DL algorithms did not perform better than the other learning algorithms.^202^ In the next study, using the DNNs, Ma et al.^185^ showed that DNNs could make better prospective predictions than RF, on large and diverse QSAR data sets. However, they could not propose a clear strategy for choosing between multitask and single‐task DNNs. Xu et al.^203^ focused on demystifying multitask DNNs and explored why multitask DNNs perform significantly better or worse for some QSAR tasks. They found that multitask DNNs can boost the predictive performance if the assistant tasks have molecules in a training set with structures similar to those in the test set of the primary task and the activities between these similar molecules are correlated. Contrarily, if the assistant tasks do not include compounds structurally similar to those in the primary task test set, multitask DNNs show no improvement in prediction, regardless of correlated or uncorrelated activities. Recently, Zakharov et al.^204^ combined multitask DNNs with consensus modeling to generate large‐scale QSAR models with improved prediction accuracy over the state‐of‐the‐art QSAR models.

Ensemble‐based ML approaches combining several basic models have also been used to overcome the weaknesses of individual learning models and thereby improve the overall performance of the QSAR predictors. There are various ensemble learning applications in QSAR predictions, including data sampling ensembles, method ensembles, and representation ensembles. Recently, Kwon et al.^205^ proposed a model that is a combined ensemble of sampling, method, and representation with an end‐to‐end NN‐based individual classifier. Their ensemble model achieved better performance than the individual models in QSAR prediction.

### 2.5 AI/ML applications in prediction of physicochemical properties and ADME‐T

#### 2.5.1 Prediction of physicochemical properties

Physicochemical properties indicate all aspects of drug action and profoundly affect the clinical success rates of drug candidates. A small molecule drug candidate must be sufficiently soluble and permeable to access its site of action and thereby engage its targets, with optimal safety profiles. Therefore, accurate prediction of the physicochemical characteristics can be beneficial for designing a new chemical entity with suitable pharmacokinetic and pharmacodynamic profiles. Researchers have adopted ML‐driven approaches to predict some key physicochemical properties, such as water solubility, membrane permeability, and lipophilicity. We provide a detailed description of each property and discuss the ML‐based techniques that specifically predict the water solubility,^206^, ^207^, ^208^, ^209^, ^210^ membrane permeability,^211^, ^212^, ^213^ and lipophilicity^214^, ^215^, ^216^, ^217^, ^218^, ^219^ in the Supporting Information. Although improved ML models have led to better prediction of molecular properties, the lack of standard criteria for performance evaluation has limited the progress. To address this, MoleculeNet, a benchmark collection for molecular ML was developed to serve as a unique resource for the scientific community to create advanced models for learning molecular properties.^154^ To further support the comparison and development of novel models, MoleculeNet has implemented various ML algorithms. Benchmark results have shown that graph convolutional network (GCN) outperforms other traditional ML methods based on molecular fingerprints and descriptors to predict molecular properties. Recent studies have supported the superior performance of GCN. Applying GCN, Feinberg et al.^155^ achieved an unprecedentedly high accuracy in predicting molecular physicochemical properties.

#### 2.5.2 ADME‐T predictions

A successful drug development pathway must include the evaluation and optimization of pharmacokinetics, pharmacodynamics, and safety profiles of a candidate molecule. In early drug discovery, evaluation of the ADME‐T properties help researchers select good drug candidates for further development. ADME‐T properties are estimated to be responsible for half of all clinical failures.^220^ In this context, in silico ADME‐T prediction models have received considerable progress over the past 40 years due to the availability of many compounds with known pharmacokinetic properties.^23^, ^221^ Prediction models usually try to build a direct relationship between a set of molecular descriptors and a given ADME‐T property.^222^ These methods represent a compound by chemical descriptors as input features such as atom counts, surface areas, weight, van der Waals volume, partial charge information, and the presence or absence of a predefined substructure. The key substructures responsible for certain toxicity are structural alerts, of which detection in given small molecules could be used for toxicity prediction.^223^ On the contrary, in these models, the toxicity properties of input compounds are HTS assay measurements of toxic effects that are highly relevant to human health, including nuclear receptor pathway assays (i.e., aryl hydrocarbon receptor, aromatase, androgen and estrogen receptor, PPAR‐gamma) and stress response pathway assays (i.e., ATAD5, antioxidant responsive element, heat shock factor response element, mitochondrial membrane potential, p53).^224^ While the conventional approaches have yielded physiologically based pharmacokinetic and pharmacokinetic‐pharmacodynamic/quantitative systems pharmacology models, researchers have applied AI/ML algorithms to produce high‐quality models with improved accuracy and thus provide meaningful predictions of ADME‐T responses using chemical structure information. For predicting regulators of drug ADME‐T properties, the classification models—DT, K‐nearest‐neighbor (KNN), SVM, RF, and NN have been extensively used. Even beyond that, the introduction of DL models has led to further developments in this area. As a good example of recent advancements in AI. ML‐aided ADME‐T prediction, Alchemite^225^—a DL model—predicts ADME‐T properties by imputing heterogeneous drug discovery data, including multitarget biochemical activities, phenotypic activities in cell‐based assays, and ADME‐T endpoints.

Moreover, the introduction of capsule networks, a new class of DNN architectures, has remarkably improved the ADME‐T prediction. To predict the cardiotoxicity of drugs, Wang et al.^226^ developed two capsule network architectures, including a convolution‐capsule network (Conv‐CapsNet) and a restricted Boltzmann machine‐capsule network (RBM‐CapsNet). Both models showed excellent performance with an accuracy of 91.8% for Conv‐CapsNet and 92.2% for RBM‐CapsNet. As the volume and chemotype coverage of the available ADME‐T databases are continually growing, we have witnessed a great progress in AI/ML‐guided ADME‐T prediction in recent years. Such advances in the field have been extensively reviewed.^136^, ^227^, ^228^, ^229^, ^230^, ^231^, ^232^

### 2.6 AI/ML applications in de novo drug design

In de novo drug design, scientists generate novel chemical entities with desired chemical and biological characteristics from scratch, aiming to achieve particular efficacy and safety profiles in a cost‐ and time‐efficient manner. Advanced AI/ML‐based tools have enabled the automated generation of new chemical entities with suitable properties. As a result of such achievements, application of AI/ML to de novo discovery has become a popular topic over the last few years. Particularly, generative molecular design based on AI/ML has aroused considerable attention. In this section, we summarize the AI/ML algorithms utilized for de novo drug design with a focus on generative models. Those who want to learn more about this subject can check other comprehensive sources in the literature.^136^, ^233^, ^234^

Traditional methods for generating novel chemical structures depend on the previously defined reaction or transformation rules, which bias the chemical space towards prior chemical knowledge. AI/ML‐based generative models are entirely data‐driven without relying on any explicit rules and can generate new molecules that are not present in a training set. Briefly, these generative models first learn from data, then create an abstract representation of the data, and finally use this representation to generate new data instances.^235^ Thus, these generative models demonstrate all aspects of an artificially intelligent system (i.e., problem‐solving, learning from experience, and coping with new situations).^235^

Recent de novo molecule‐generative models with an ML structure include adversarial autoencoders (AAE),^236^, ^237^, ^238^ variational autoencoders (VAE),^239^, ^240^ and recurrent neural networks (RNN).^241^, ^242^, ^243^, ^244^ In generating novel molecules represented by simplified molecular input‐line entry system (SMILES) strings, RNN is a promising approach for learning from large sets of SMILES strings and generating ligands with similar activities to those of the training set templates, but with novel scaffolds. However, the percentage of valid SMILES, internal diversity, and the similarity of molecules to the training data set in the libraries generated by any given approach have been a matter of debate. To address these issues, Reinforcement learning (RL) has been embedded in ML architectures.^239^, ^245^, ^246^, ^247^, ^248^ Introducing a task‐specific reward function, RL‐assisted models are able to produce chemically feasible and predominantly novel molecules with appropriate molecular properties. For the generation of novel small molecules with the desired characteristics, generative adversarial networks (GAN) also have been employed. For example, druGAN^237^ (drug‐generative adversarial network) has been developed for producing new molecules with specific anticancer properties.

Another commonly used drug design approach is to generate new analogs/similar drugs of a given set of drugs. In such cases, the transfer learning models have been integrated into NN architectures to increase the prediction accuracy by taking knowledge acquired from training on a previous problem and applying them to a new but related problem.^249^, ^250^

In the generative drug design models above, many ML architectures use the SMILES as molecular representation. SMILES provides a linear representation, referred to as a SMILES string that can be translated into a graph and enables a straightforward application. However, it has one or more limitations: Generated SMILES may not represent a chemically feasible structure, and even a single character alteration in a SMILES representation can change the underlying molecular structure significantly.^251^ To overcome its limitations, researchers proposed several solutions like converting SMILES strings into a new SMILES‐like syntax^252^ or utilizing grammatical evaluation of the SMILES syntax.^253^ Besides the SMILES string representation, molecular graphs have also been used to train ML‐based molecule generation algorithms.^254^ In molecular graph generators, structures are directly represented as graphs in every step and substructures are inferred from the partially generated molecular graphs.^255^ Examples of such ML models to design de novo molecules based on graph representation includes GANs^256^, ^257^ and VAEs.^258^, ^259^

In addition to the models mentioned above, some AI/ML‐driven de novo molecule design tools are distinguished by introducing novel approaches. An automated de novo molecular design tool, DINGOS,^260^ has been developed to emulate the approach of a synthetic chemist. It assembles drug‐like new compounds through modular and synthetically feasible design schemes, considering the synthetic feasibility of each step. In brief, the DINGOS algorithm combines a rule‐based approach with an ML model trained on known successful synthetic routes, while the former ensures the synthesizability and the later provides a directed approach to limiting the output molecules to compounds with desirable similarity to the template. Another remarkable ML‐based generative approach is proposed by Méndez‐Lucio et al.,^261^ which bridges systems biology and molecular design. To our knowledge, it is the first AI/ML‐based drug design tool that combines transcriptomic and structural data. Conditioning a GAN architecture with compound‐induced transcriptomic data (i.e., L1000 data set), they can automatically design molecules that potentially produce the desired transcriptomic outcome. Their model allows the design of active‐like molecules for a desired target using just gene expression signature of target perturbation. However, the current version is not capable of generating compounds that can reverse disease‐related gene expression signatures. Also, its performance has not been evaluated in a real drug‐discovery setting yet.

Among all the studies of AI/ML‐based generative molecular design, maybe the most‐mentioned^262^ one is published by Insilico Medicine,^263^ showing how AI for generative chemistry can be used to drive rapid drug discovery. The goal of the study was to demonstrate that efficacious drugs can be developed in just 21 days for a new target. For this purpose, they have developed a generative tensorial reinforcement learning (GENTRL) model, which can be seen as an advanced version of their earlier algorithms on VAE^238^ and GAN,^247^ to design DDR1 kinase inhibitors. Notably, this study has two major limitations: First, DDR1 is considered to be the most promiscuous kinase^264^; thus, developing compounds targeting this protein may be considered low hanging‐fruit. Second, the seemingly novel compound is highly similar to the widely used cancer drug ponatinib, indicating the limitation of the approach^265^ in assessing truly novel scaffolds. Therefore, there is still room for improvement of AI/ML‐inferred small molecules to obtain a clinical candidate.

### 2.7 AI/ML applications in prediction of drug sensitivity and response

Personalized drug response prediction aims to improve the targeted therapy response in complex diseases like cancer.^266^ However, the limited application of candidate drugs in clinical settings and the heterogeneity among cancer patients make it difficult to tailor therapy for each individual cancer patient. Personalized treatment design requires predictive methods that are capable of exploiting large, heterogeneous, and sparsely sampled data sets. Accurate AI/ML‐based models employing in vitro and in vivo data sets have the potential to improve the prediction of response of cancer cells to a given compound. There are various AI/ML models to predict drug sensitivity and anticancer drug response. In such efforts, elastic net regression,^267^, ^268^, ^269^ ensemble‐based approaches,^270^, ^271^ transfer learning,^272^ autoencoders,^266^, ^273^, ^274^, ^275^ and multitask learning approaches^276^, ^277^, ^278^ have been widely used. The details about these AI/ML applications can be found in the Supporting Information.

### 2.8 AI/ML applications in prediction of drug–drug interactions

In the treatment of complex diseases such as neurological disorders, diabetes, cancer, or cardiovascular disease, drug combinations are highly utilized for medical intervention. Coadministration of drugs in the treatment aims to enhance efficacy, reduced toxicity, and prevent the emergence of resistance. Drug combinations are classified as synergistic, antagonistic, or additive. Drug synergy is the interaction of two or more drugs, causing the total effect of drugs to be greater than sum of individual effects of each drug.^279^ If drugs act synergistically, lower doses of each drug could potentially be enough to provide the desired outcome allowing for less adverse effects. Opposite to synergism, the antagonistic combination means that the combined activity of the drugs is lower than the response of the individual agents.^280^ Finally, a drug combination is considered to be additive when the response of each drug neither masks nor enhances the efficacy of others.^281^ Although combinatorial therapy has advantages over monotherapy, developing a new drug combination regimen that can be transferred to the clinic is still challenging. So far, the effective drug combinations have been suggested based on either clinical experience or HTS of drug pairs at different concentrations on cell lines. However, the former involves the risk of harm to patients, and the latter is unfeasible to test the complete combinatorial space.^282^ To accelerate conventional combinatorial therapy efforts, AI/ML algorithms have begun to be utilized for prioritizing the drug pairs and exploring the larger combinatorial space. Tonekaboni et al.^283^ introduced some examples of various ML‐based prediction frameworks for drug–drug interactions. To avoid duplication, we overview the AI/ML applications in combinatorial therapy after that time, including the applications in cancer^284^, ^285^, ^286^, ^287^, ^288^ and depression treatment,^289^ antimalarial,^290^ and antibiotic^291^ discovery, along with the available AI/ML‐based tools to predict the synergistic effects of drug combinations^292^, ^293^, ^294^ in the Supporting Information.

In addition to the synergistic effects, drug–drug interactions can induce unexpected adverse drug reactions. Such adverse reactions caused by drug–drug interactions could lead to death in some extreme cases.^295^ Therefore, AI/ML‐based models have been developed to predict the risk of side effects due to drug–drug interactions. Applications of GCN,^296^ DNN,^297^ and ML architectures^298^ showed promising results for predicting adverse drug reactions of drug combinations. Lee and Chen^299^ extensively discussed the role of ML approaches in detection and classification of side effects caused by drug–drug interactions in their review of previous studies. In a recent study, Shankar et al.^300^ predicted the adverse drug reactions of coadministered drug pairs using an ANN trained on transcriptomic data, compound chemical fingerprint, and Gene Ontologies.^300^

### 2.9 AI/ML applications in drug repurposing

Drug development and trials in animals and humans is a time‐consuming and expensive process. In general, the whole process for developing a new FDA‐approved drug requires 10–17 years of period and the tremendous cost of $2.6 billion.^301^ However, high expenditures for drug development has not been able to increase the rate of approved drugs.^302^ Among the reasons for this limited approval rate, a key factor is the continued adherence to the classical “one gene, one drug, one disease” paradigm in the traditional drug development.^303^ Since drug targets do not operate in isolation from the biochemical system, each DTI must be studied in a broader integrative context.^304^ This approach provides new insights into “off‐target” effects (i.e., side effects), resistance to precision therapy, and drug mechanism of action that can inform drug‐repurposing efforts.

Drug repurposing, also known as drug repositioning, denotes the new indications of existing drugs and is an alternative over the de novo drug development. Although the unknown underlying complex biology and pharmacology has challenged the drug‐repurposing attempts, intelligent computer algorithms offer a strategy for detecting potential drug indications by integrating large‐scale heterogeneous data (i.e., genomic, transcriptomic, phenotypic, chemical, and bioactivity) from hundreds of approved drugs. Various specially designed AI/ML models have been proposed for detecting novel drug indications. Here, we classify the ML applications for drug repositioning into the following three categories: (i) Similarity‐based methods that employ different types of classifiers like logistic regression,^305^, ^306^ SVM,^307^, ^308^, ^309^ RF,^310^, ^311^ KNN,^312^ and CNN,^313^ (ii) feature vector‐based methods that utilize supervised^314^, ^315^, ^316^, ^317^, ^318^ and semisupervised^319^, ^320^, ^321^ learning algorithms, and (iii) network‐based methods that mainly use semisupervised learning algorithms (e.g., Laplacian regularized least square,^322^, ^323^, ^324^ label propagation,^325^ random walk,^326^ and RF^310^). We provide an in‐depth discussion of these three classes of AI‐based drug repositioning applications in the Supporting Information. Particularly, in early 2020, researchers at MIT published a milestone paper using a DL approach to antibiotic discovery.^153^ They trained the deep GCN model based on molecular features and predicted *halicin* as an antibacterial molecule from the Drug‐Repurposing Hub. Halicin showed a broad‐spectrum activity against drug‐resistant strains in mice. This is the first time an AI/ML‐assisted tool was used to identify thoroughly new types of antibiotic from scratch, without the need for any previous human assumptions.

## 3 AI/ML APPLICATIONS IN CNS DRUG DISCOVERY

**Figure 4:**
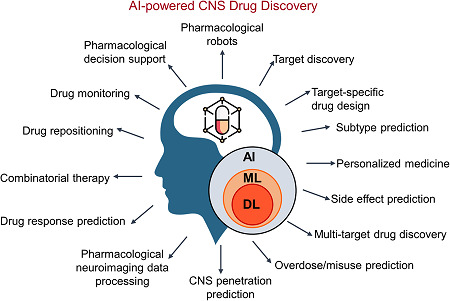
AI/ML‐enabled improvements in the treatment of CNS diseases. DL is a subset of ML, which is a subset of AI and their applications address a wide range of challenges in CNS drug discovery and development. The application fields portrayed here are discussed in the Section 3. AI, artificial intelligence; CNS, central nervous system; DL, deep learning; ML, machine learning

CNS diseases are a group of neurological disorders that impose a significant economic and social impact. Development of new drugs for CNS diseases poses unique challenges compared to other diseases, including the complexity of brain anatomy and function, incomplete understanding of the biology of the complex nature of CNS diseases and the presence of BBB. In this section, we present an overview of AI/ML‐based approaches to meet challenges such as BBB permeability in CNS drug discovery (Figure 4).

### 3.1 BBB permeability prediction

Despite significant progress in our understanding of CNS diseases, the development of novel therapies for CNS diseases faces some great challenges. In addition to the difficulties in CNS target identification, designing new molecules with the ability to penetrate the BBB is also a major obstacle. The role of the BBB is to protect the brain from variations in blood composition (e.g., hormones, amino acids, and potassium) and circulating pathogens. It consists of capillary endothelial cells that are lined by the basal lamina made from structural proteins (i.e., extracellular matrix proteins collagen and laminin), pericytes, astrocytic endfeet, and microglial cells.^327^ This biologic membrane allows the uptake of water, glucose, and essential amino acids, the efflux of small molecules and nonessential amino acids from the brain to the blood and the passage of some molecules by passive diffusion.^328^ While negligible penetration is desirable to minimize the brain side effects for peripheral drugs, high penetration is needed for CNS‐active drugs. To improve success rates in CNS drug discovery, the BBB permeability of drug candidates needs to be addressed early in the drug discovery process.

In recent years, AI‐based predictive models have been proposed to minimize the number of laborious, expensive, time‐consuming BBB permeability experiments that need be carried out in CNS drug discovery. For the construction of BBB permeability predictive models, researchers have employed various supervised learning approaches, such as SVM,^222^, ^329^, ^330^, ^331^, ^332^, ^333^ recursive partitioning (RP),^334^, ^335^ Gaussian process,^336^ DT,^337^ KNN,^338^ linear discriminant analysis,^339^ consensus classifier,^340^ and ANN.^341^, ^342^, ^343^ All of these methods were developed to process physical and chemical features, which mainly include molecular weight, hydrophilicity (ClogP), lipophilicity (ClogD), topological polar surface area, acidic and basic atoms numbers, hydrogen bond donors and acceptors, water‐accessible volume, flexibility (rotatable bonds), van der Waals volume, and ionization potential.

The predictive capability of all the methods mentioned above is limited to passive diffusional uptake and predominantly relies on few molecular descriptors. However, many molecules, for example, glucose and insulin, pass BBB via complex mechanisms that involve specific drug‐transporter/drug‐receptor interactions.^344^, ^345^ Hence, such mechanisms are hard to be described by simple physicochemical features of compounds. Moreover, achieving therapeutic drug concentrations in CNS may be limited by membrane transporters such as the ATP‐binding cassette and efflux transporter P‐glycoprotein (P‐gp),^346^ which mediates efflux of drugs from the BBB. Although the primary role of these efflux transporters is limiting the brain entry of neurotoxins, they also limit the entry of many therapeutics and may contribute to CNS pharmacoresistance.^347^, ^348^ Therefore, prediction methods need to both overcome the limitations of physicochemical features and address the multiple mechanisms associated with the drugs that pass the barrier and sustain in the brain. For this purpose, Yuan et al.^333^ developed an SVM model by combining physicochemical properties and molecular fingerprints: The former is related to passive diffusion while the latter is associated with specific interactions, such as uptake, efflux, and protein binding. When compared to other SVM‐based BBB permeability predictors, the improved accuracy of their model shows that integration of the physicochemical properties and fingerprints can yield better predictions. Actually, all the AI/ML‐based models we have mentioned so far have been trained only on molecular properties disregarding the other types of information related to the efficacy of CNS drugs.

Clinical trials of many drug candidates generate a large amount of phenotypic data in CNS, but the relationship between the CNS side‐effects of drugs and their BBB permeation has not been adequately captured. To bridge the knowledge gap, Gao et al.^349^ developed a BBB permeability prediction tool utilizing drug clinical phenotypes (drug side effects and drug indications). Although they explored the BBB permeability prediction from a new angle by accounting for passive diffusion as well as putative contributions of active transport and other complex mechanisms, the accuracy of their SVM method still needs to be improved. In fact, the features based on physics and chemistry are different; hence, the relation between drug side effects and therapeutic effects is more abstract and deeper.^350^ For this reason, classical classification algorithms are not able to efficiently explore the relationship between data and results. On the contrary, DL architectures have the ability to extract useful information from complex data structures with abstract relationships. Therefore, Miao et al.^350^ built a DL model to predict the BBB permeability of drugs based on clinical features and achieved better performance than the other existing methods.

### 3.2 AI/ML applications in drug discovery for neurological disorders

#### 3.2.1 AI/ML applications in drug discovery for neurodevelopmental disorders

Schizophrenia is arguably the most puzzling of psychiatric disorders.^351^ As a neurodevelopmental disorder,^352^ schizophrenia shows a lifetime prevalence of 0.30%–0.66%,^353^ generally beginning before age 25 years and persisting throughout life, making it one of the leading factors of global disease burden.^354^ Despite more than a century of research, its complex pathophysiology remains unknown,^355^ and currently, there is no effective drug for schizophrenia. Therefore, there is a need for alternative strategies to develop innovative drug treatments for schizophrenia.^356^ In recent years, AI/ML has seen as a promising technology to inform schizophrenia diagnosis,^355^, ^357^ detecting heterogeneity,^358^, ^359^, ^360^ subtyping,^361^, ^362^ and treatment.

In drug discovery studies for schizophrenia, researchers have utilized AI/ML methods with various purposes, including drug target identification,^363^, ^364^ developing QSAR models,^365^ predicting monitoring dosing compliance,^366^ predicting GPCRs targeting compounds,^364^ and drug repositioning.^367^ Specifically, schizophrenia target genes were identified based on publicly available microarray data sets using an SVM‐RFE (recursive feature elimination)‐based feature selection, where the genes initially ranked by an SVM classifier and the signature was then identified by discarding the genes that were not differentially expressed. To detect optimal biomarkers of presynaptic dopamine overactivity, which may cause schizophrenia, an SVM classifier was used.^363^ SVM classifiers were also used to predict QSAR models of the GABA (gamma aminobutyric acid) uptake inhibitor drugs, which can be beneficial in the treatment of schizophrenia.^365^ Moreover, SVM outperformed the other ML methods in predicting the repositioning drugs for schizophrenia when trained on drug expression profiles.^367^ On the contrary, for schizophrenia subtyping, an unsupervised learning approach, multi‐view clustering, was employed by combining transcriptomic data with clinical phenotypes.^368^ Setting a good example of the beneficiary of AI/ML in clinical drug trials, a novel AI platform AiCure^366^ on mobile devices was used to assess the dosing compliance in Phase 2 clinical trial in schizophrenia patients. It, simply, confirms the medication ingestion visually by using facial recognition and computer vision.

One of the major obstacles in developing AI/ML methods for schizophrenia drug discovery is data availability.^369^ Publicly available, large‐scale, well‐structured information on neural phenotypes, genomics, and clinical stages are greatly lacking, which arouses questions for the generalizability of AI/ML algorithms across different data sets without performance loss. However, the availability of such integrative databases can encourage the development of AI/ML‐based methods to investigate personalized therapies by solving the disease heterogeneity.

Another neurodevelopmental disorder is autism spectrum disorder (ASD), which is characterized by deficits in social communication and social interaction and the presence of restricted, repetitive patterns in behaviors or interests.^370^ ML methods have been utilized in ASD research for improving the diagnosis^371^ and prognosis prediction.^371^ Also, there are few ML applications in drug discovery for ASD. For example, ML‐based cluster analysis (i.e., affinity propagation and k‐medoids) of clinical data (i.e., signs and biomarkers) exhibited a good performance in drug response prediction of ASD patients.^372^ Moreover, Bayesian ML models trained on HTS data revealed the potential repurposing of nicardipine or other dihydropyridine calcium channel antagonists for the treatment of Pitt Hopkins Syndrome, a rare genetic disorder that exhibits features of autistic spectrum disorders.^373^ Recently, ML algorithms have been employed to predict the functional effects of variants in voltage‐gated sodium and calcium ion channels, which have been associated with ASD, schizophrenia and developmental encephalopathy.^374^ Being trained on sequence‐ and structure‐based features, the ML model predicted the gain or loss of function effects of likely pathogenic missense variants in ion channels and the results were validated in exome‐wide data. On the contrary, the toxic compounds may trigger the recent increases in neurodevelopmental disorders among children.^375^ To identify developmental neurotoxicants, researchers developed ML algorithms to predict the neurodevelopmental toxicity of compounds.^376^, ^377^

#### 3.2.2 AI/ML applications in drug discovery for depression

AI/ML‐based methods have been utilized in psychiatric drug discovery, especially for pharmacological decision support.^367^, ^378^, ^379^ In a depression study, researchers have developed a gradient boosting machine using the predictors identified by the elastic net to predict whether a patient will achieve symptomatic remission using an antidepressant, citalopram.^380^ This model was also successfully applied to an escitalopram treatment group of an independent clinical trial.^378^ In the next study of Chekroud et al.,^381^ they clustered the symptoms using an unsupervised learning approach (hierarchical clustering) and predict the responsiveness of each cluster to the treatment of different antidepressant drugs using the same model in the previous study. To provide decision support for clinicians to select the best drugs for a given cluster of symptoms, a web‐based application was designed. This AI‐based service is prospectively tested in hospital settings and thereby serve as a promising model for direct research translation.^382^

On the contrary, the model of Chekroud et al.^380^ has some limitations. The model only predicts whether a patient responds to a specific antidepressant without measuring the degree of antidepressant response. Since it was designed for only one antidepressant, the model is not capable of selecting the most effective drugs among various antidepressant candidates for patients.^383^ To address these limitations, Chang et al.^383^ developed an Antidepressant Response Prediction Network (ARPNet) model based on an NN architecture. Through the literature‐based and data‐driven feature selection process, ARPNet predicts the degree of antidepressant response, whether the patient will reach clinical remission from depression, and a patient's response to a combination of one or more antidepressants.

Electroencephalography (EEG) and functional magnetic resonance imaging (fMRI) data also have been employed in predicting drug responses to treatments of depression. Zhdanov et al.^384^ used an SVM classifier to accurately predict the outcome of escitalopram treatment using patients' EEG data at the baseline and after the first 2 weeks of treatment. To identify a robust signature from resting‐state EEG that would predict response to antidepressants, Wu et al.^385^ designed an end‐to‐end prediction algorithm with a latent space model. They applied their algorithm, Sparse EEG Latent SpacE Regression (SELSER), to data from an imaging‐coupled, placebo‐controlled antidepressant study and identified an EEG signature of patient's response to antidepressant treatment (i.e., sertraline). Ichikawa et al.^386^ aimed to develop a melancholic depressive disorder biomarker to extract critically important functional connections (FCs) from fMRI data. By combining two ML algorithms (i.e., L1‐regularized sparse canonical correlation analysis and sparse logistic regression), they developed a classifier for melancholic depressive disorder and found out that antidepressants had a heterogeneous effect on the identified FCs of melancholic depressive disorder.

Although some of the recent AI/ML‐aided tools have been rapidly translated into the clinical trials, the AI/ML methods still are not used widely in clinical practice, while AI has been employed in psychiatric research over 20 years.^387^ To close the gap between research and clinic, we need to improve the validity of diagnostic and prognostic labels, representability of the features, and generalizability of models.^388^ As scientists continue to work to bridge the gap between research and clinic, it will be possible to provide efficient, personalized treatments based on a patient's unique characteristics.^389^

#### 3.2.3 AI/ML applications in drug discovery for Parkinson's disease

Parkinson's disease (PD) is the second most common age‐related neurodegenerative disorder, affecting over 1% of the population above the age of 60, increasing to 5% in individuals above 85 years of age.^390^ PD is a prime example of a multifaceted disease, including a broad range of motor and non‐motor symptoms and possible contribution of genetic and environmental risk factors.^391^ Currently, there is no treatment to prevent the progressive depletion of dopaminergic neurons in the substantia nigra that underlies the movement control and cognitive loss, which is manifested with tremors and memory loss.^392^, ^393^ Available drug treatments are based on the administration of levodopa (l‐dopa) and catechol‐O‐methyltransferase or monoamine oxidase B inhibitors, offering only symptomatic relief to the patients.^392^

In PD research, previous AI applications have focused on diagnostic biomarker discovery in cerebrospinal fluid (CSF) and blood^394^, ^395^, ^396^, ^397^ and remote monitoring of treatment response by using electronic wearables.^398^, ^399^, ^400^, ^401^, ^402^ On the contrary, recently, AI/ML has received little attention in PD drug discovery. Particularly, Shao et al.^403^ initially built SVM models to quickly select the compounds containing indole–piperazine–pyrimidine scaffold among large chemical databases and subsequently identified novel compounds that simultaneously bind the two receptors—adenosine A2A receptor and dopamine D2 receptor—implicated in the PD pathophysiology. In another study, Sebastián‐Pérez^404^ utilized several ML techniques to infer QSAR models for the identification of putative inhibitors of LRRK2 protein, a key genetic risk factor for familiar and sporadic PD. Moreover, AI‐based technologies have helped overcome the drug side effects in PD treatment. While l‐dopa has remained the cornerstone of PD therapy for reducing the symptoms associated with dopamine deficiency, almost half of PD patients treated with it eventually develop levodopa‐induced dyskinesia (LID), a side effect that causes abnormal involuntary movements. In a review paper, Johnston et al.^405^ discussed the use of AI platforms to identify repurposing candidates for LID treatment and highlighted the potential of AI approaches by designing a drug repositioning case study. To identify novel repurposing candidates that may reduce LID, they utilized a literature mining approach based on an IBM Watson engine, where the semantic similarity and a “graph diffusion” algorithm were applied to score and rank each candidate drug.

Along with the identification of novel and repurposing candidates, AI/ML techniques have been applied to the development of in vitro and in vivo PD models for drug screening. Monzel et al.^406^ created a human midbrain organoid model of PD as an in vitro toxicity assay and built an RF classifier to predict compounds' neurotoxic effect on organoids based on cellular features. To establish an efficient drug‐testing route, Hughes et al.^407^ developed a zebrafish model of PD together with an AI/ML‐based method to classify movement disorders in this model using high‐resolution video captures. Encouraging results of all the studies discussed above, highlight the potential benefits of AI/ML applications for the discovery of efficient and multitargeting drugs against emerging targets in PD as well as the screening of the drug effects on PD models.

#### 3.2.4 AI/ML applications in drug discovery for Alzheimer's disease

Increasing life expectancy has produced a dramatic rise in the prevalence, and thus impact, of aging‐related diseases. The most prevalent neurodegenerative disease in older adults is Alzheimer's disease (AD), characterized by insidious and progressive impairment of behavioral and cognitive functions, including memory.^408^ The cause of AD is still unclear; however, generally accepted neuropathological hallmarks of AD include extracellular A‐beta plaques and intracellular neurofibrillary tangles, along with neuronal and synaptic loss and/or dysfunction.^409^ Current drugs for AD target cholinergic and glutamatergic neurotransmission, thus improving symptoms, although they show limited benefits to most AD patients.^410^ Therefore, new treatments are urgently needed to prevent or delay disease onset, slow its progression, or improve patients' symptoms.^411^ However, drug development for AD has been extraordinarily difficult, with a failure rate of over 99% and no new drug approved since 2003.^411^ AD drug failures are likely due to the lack of sufficient target engagement and toxicity, while drug discovery efforts mainly challenged by an incomplete understanding of AD pathogenesis, multifactorial etiology, and complex pathophysiology.

In recent years, AI/ML‐based models have become popular in AD research, mostly utilizing for AD diagnosis and prognosis in dealing with electronic health records and images.^412^ On the contrary, AI/ML techniques have not been widely employed in AD drug discovery. However, there have been a few studies that show the potential benefits of AI/ML applications for the discovery of AD drugs. ML approaches have assisted the target identification and characterization in AD, which is the initial phase of drug discovery. For example, Cordax^413^ (https://cordax.switchlab.org) is a novel structure‐based amyloid core sequence prediction method that implements ML to detect aggregation‐prone regions in proteins as well as to predict the structural topology, orientation and overall architecture of the resulting amyloid core. As an aggregation predictor, it uses structural information on amyloid cores currently available in the protein databank and translates structural compatibility and interaction energies into sequence aggregation propensity using logistic regression. Along with the characterization of amyloid fibrils, ML approaches have been utilized for identifying potential drug targets. HENA,^414^ a heterogeneous network‐based data set for AD, integrates distinct data types (i.e., PPI, gene coexpression, epistasis, genome‐wide association study, gene expression in different brain regions, and positive selection data) through GCN to predict AD‐associated genes.

Researchers have built ML models—SVM, ANN, and RF—to predict the inhibitory effect of compounds against AD‐related proteins—histone deacetylase (HDAC),^415^ acetylcholinesterase (AChE),^416^ and S100 calcium‐binding protein A9 (S100A9),^417^ respectively. Although these target‐specific models were successful for predicting the bioactive compounds, a high level of reliability is necessary for prioritizing compounds that are ultimately translated into assays. To generate hyper‐predictive ML models, Jamal et al.^418^ have included dynamic properties of compounds and protein–ligand interactions. Extracting the dynamic descriptors from molecular dynamics simulations of caspase‐8 ligand complexes to train ANN and RF models, they predicted the active compounds against caspase‐8, which plays a key role in causing AD. The major challenge in developing such predictive models of inhibitor activity is the lack of data on true‐negative compound–protein interactions. To address this challenge, Miyazaki et al.^419^ constructed a graph CNN model to explore compounds specifically targeting proteins without using the information on the true‐negative interaction and applied the model to identify inhibitors of BACE1 enzyme, a major target for AD.

Although these ML applications have advanced the discovery of single‐target inhibitors, the complex nature of AD requires the discovery of multitarget drugs to address the multiple pathways contributing to disease pathogenesis. Therefore, researchers have developed ML algorithms for predicting multitarget‐directed compounds against AD. Kleandrova et al.^420^ designed seven molecules as triple target inhibitors of AD‐related proteins, namely GSK3B, HDAC1, and HDAC6 by combining perturbation theory and ML‐based on ANN. Using a new multitask QSAR model based on the linear discriminant analysis, Concu et al.^421^ predicted the inhibitors of the two isoforms of the monoamine oxidase (MAO) enzymes, MAO‐A and MAO‐B, which are involved in the pathology of AD, PD, and other neuropsychiatric disorders. As epigenetic therapeutics for AD, HDAC inhibitors have shown promise; however, nonspecificity and nonselectivity are the major problems of current HDAC inhibitors. Therefore, Gupta et al.^422^ combined VS and ML to classify the HDAC inhibitors and identified a novel compound that potentially inhibits all isoforms of class I and class IIb HDAC for AD therapy. In addition to these, Fang et al.^423^ built 100 binary classifiers based on the naive Bayesian and RP algorithms to predict active small molecules against 25 key targets toward AD. Experimental validation of the predicted molecules yielded a compound that is a dual cholinesterase inhibitor and H3R antagonist. In their following study,^424^ the system has been updated by assembling 204 binary classifiers towards 54 critical targets related to AD and the information of the classifiers was shared in a web server named AlzhCPI. Utilizing this classifier system, another group of researchers^425^ has identified multiple targets of a traditional Chinese herbal medicine formula, Naodesheng, for application to AD. Natural products has continued to generate an increased interest as a mean of discovering novel bioactive compounds against AD. Grisoni et al.^426^ proposed a VS protocol based on ML models to explore the bioactive synthetic mimetics of the natural product galantamine, which is the first natural product‐based AD drug approved by the FDA in 2001.^427^ Using an ML‐based selection and target profiling program, they identified galantamine‐mimetic small molecules with multitarget activity on enzymes and receptors related to AD.

Besides the predictions of multitarget compounds based on their bioactivity against known drug targets in AD, Jamal et al.^428^ predicted small molecules that show a high binding affinity for ML‐inferred possible therapeutic targets. Unlike previous studies that target known AD‐related proteins, they initially predicted the probable AD‐associated genes using ML classifiers that are trained on network, sequence and functional features. Then, they used a conventional VS tool to select the compounds that have high affinity for the majority of the predicted targets.

In addition to applications for identifying small molecules towards therapeutic targets for AD, ML techniques also have been utilized in drug repositioning efforts. For example, telmisartan has been associated with AD by a network‐based classification model.^310^

AI/ML approaches have also been applied to drug response studies to treat AD patients in a more precise, personalized way. Hampel et al.^429^ has built an AI/ML‐based precision medicine framework for identifying the genomic biomarkers of response to AD therapy. Specifically, they studied blarcamesine (ANAVEX2‐73), a selective sigma‐1 receptor agonist, in a Phase 2a trial, where they obtained the patients' whole‐exome and transcriptome data and recorded the measures of safety, clinical features, pharmacokinetics, and efficacy. They analyzed the relationship between the patient data and efficacy outcome measures using unsupervised formal concept analysis, which ultimately identified the biomarkers of drug response. On the contrary, Lu et al.^430^ evaluated the therapeutic effects of Dengzhan Shengmai formula, a traditional Chinese medicine, on AD patients by analyzing the diffusion tensor imaging data with ML. Their ML classifier revealed significant white‐matter network alterations after treatment.

#### 3.2.5 AI/ML applications in anesthesia and pain treatment

The CNC drugs include general anesthetics and the analgesics, as well. In the past few years, we have witnessed the widespread use of autonomous and AI‐based recommender systems in therapeutic decision making in anesthesia and pain management. Especially, pharmacological robots have become an integral part of the anesthesia field, offering a personalized anesthetic drug dosage for maintaining patient homeostasis during general anesthesia and sedation.^431^ These robots use complex ML algorithms based on patient data (e.g., EEG monitor, blood pressure, heart rate, etc.) and pharmacokinetic features of drugs to provide the optimal drug dosage. The role of pharmacological robots and even more intelligent autonomous systems (i.e., cognitive robot, which can recognize crucial clinical state that requires human intervention) in the anesthesia field has been comprehensively overviewed by Cédrick et al.^432^ Besides the robotic systems, ML applications assisted the clinicians^433^ to monitor the drug‐specific anesthetic states^434^, ^435^, ^436^ and predict the adverse outcomes in anesthesia patients.^437^, ^438^, ^439^

Similar to the anesthesia field, AI models have mainly utilized for clinical decision support in pain management. With the increasing amount of data collected by state‐of‐the‐art monitoring sensors and the Internet of Things, the AI‐assisted patient‐controlled analgesia has a great potential for personalized pain therapy.^440^ The other clinical applications of AI systems in pain management include prediction of pain severity/modality and analgesic requirements,^441^, ^442^, ^443^ individualized medicine decision support in analgesic treatment,^444^, ^445^ prediction of the effectiveness of the analgesics,^446^, ^447^ and prediction of medication overuse.^448^, ^449^, ^450^ Besides the clinical applications, researchers have employed ML methods at the early stages of analgesic discovery, such as identifying novel genes and pathways associated with acute and chronic pain^451^ and predicting inhibitors of a drug target for pain (i.e., NaV1.7 sodium channel).^452^ To facilitate the prediction of novel multi‐target analgesics or drug combinations for pain treatment, researchers have established a comprehensive pain‐domain‐specific chemogenomics knowledgebase that includes the analgesics in current use, pain‐related targets with all available 3D structures, and the compounds reported for these target proteins.^453^

## 4 CONCLUSIONS AND FUTURE DIRECTIONS

Given the complexity of neurological disorders, CNS drug development is still a long, expensive, inefficient, and challenging process with a low rate of new successful therapeutic discovery. To overcome the challenges of CNS drug discovery, researchers have utilized AI/ML‐based methods, which have played a promising role in all stages of drug discovery for a variety of diseases (Table 2). In general, AI/ML practices in pharmaceutical development have aroused great interest among researchers working in academia and industry. The number of start‐ups in this area has grown rapidly and reached 230 by June 2020.^454^ Also, many pharmaceutical companies have invested in internal AI‐based research programs as well as in collaboration with AI start‐ups and academic institutions.^455^, ^456^ Recently, we have witnessed a massive collaborative effort by both academia and industry in response to the COVID‐19 outbreak. Labs and AI firms have shared their data and pipelines in open‐sourced platforms. For example, Google Deepmind has released the 3D structures of SARS‐CoV‐2 proteins that have been predicted by their AlphaFold system. Although AI‐enabled solutions have emerged as a crucial tool for transforming the process of therapeutic development, the use of AI technologies to improve CNS drug discovery is still at an early stage. Below, we discuss the limitations as well as the future directions to guide further advancement in this evolving field.

**Table 2:** The promise of AI/ML‐based drug discovery strategies in CNS disorders

Application field	Schizophrenia	Autism spectrum disorder	Depression	PD	AD	Anesthesia	Pain treatment
Diagnosis/prognosis	✓	✓	✓	✓	✓	‐	✓
Subtyping	✓	‐	‐	‐	‐	‐	‐
Heterogeneity detection	✓	‐	‐	‐	‐	‐	‐
Target identification	✓	‐	‐	‐	✓	‐	✓
Inhibitor discovery	✓	✓	✓	✓	✓	‐	‐
Multitarget drug discovery	‐	‐	‐	‐	✓	‐	✓
Drug repositioning	✓	✓	✓	✓	✓	‐	‐
Drug response	‐	✓	✓	‐	‐	‐	‐
Variant effect	‐	✓	‐	‐	‐	‐	‐
Developmental neurotoxicants	✓	✓	‐	‐	‐	‐	‐
Pharmacological decision support	‐	‐	✓	‐	‐	✓	✓
Drug response monitoring	‐	‐	‐	✓	‐	✓	‐
Adverse drug effects	‐	‐	‐	✓	‐	✓	‐
Drug screening	‐	‐	‐	✓	‐	‐	‐
Overdose and misuse	‐	‐	‐	‐	‐	‐	✓

Abbreviations: AD, Alzheimer's disease; AI, artificial intelligence; CNS, central nervous system; ML, machine learning; PD, Parkinson's disease.

The main bottleneck in applying AI/ML into CNS drug discovery is the lack of high‐quality, well‐annotated data sets to train effective algorithms. The data collected in the public databases are generally generated by different biological assays, methods, or conditions, which are not comparable. Also, multiple data sets on the same subject may contradict each other. Therefore, filtering the raw inputs to obtain high‐quality data is a crucial step before performing specific AI/ML tasks.

The “black box” nature of most next‐generation AI architectures an additional challenge in CNS drug discovery. The lack of interpretability of AI/ML‐generated results limits their applications. While this is not the case for simpler ML models (i.e., XGBoost, TensorFlow, Lasso, Ridge, Elastic Net), for more advanced ML models (i.e., DNN) the internal workings remain a mystery. Hence, researchers cannot explain how the model arrives at the result and understand the underlying biological mechanisms. This also makes it more difficult to troubleshoot these models when they unexpectedly fail. Therefore, there is a critical need to develop methods for decoding the black boxes of DL.

Also, the amount of the available data directly affects the performance of AI/ML models, since successful training of algorithms relies on suitably large amounts of training data. This is a particular challenge in nominating new targets or drugs for many neurological conditions for which no treatment reverses the disease state. When there are not sufficient training examples for performing a drug discovery task, transfer learning technology, which learns from one task and applies it to the other task, can offer a solution. However, in the long term, the most promising solution to overcome data scarcity would be for the scientific community to share their data. Such large‐scale sharing of data would make significant improvement in the CNS drug discovery process, with advances in hardware that lead to faster machines such as quantum computers in the near future.

A particular limitation for the AI/ML applications in CNS drug discovery is the unknown pathophysiology for many nervous system disorders, which makes target identification very challenging. To explore the complex disease mechanisms and define the right biological targets, we need better AI/ML tools that can pull information out of the data sets generated across the different biological layers (e.g., transcriptomics, proteomics, and metabolomics). Here, capsule networks,^457^ a next‐generation AI architecture where CNNs are encapsulated in an interconnected module, can provide a solution. As the first application of capsule networks to drug discovery, capsule networks showed excellent performance to predict the cardiotoxicity of compounds, which highlights their unique potential in drug discovery efforts.^226^ Because of the modular representation of the CNNs, capsule networks can learn from heterogeneous data sets by preserving the hierarchical aspects of the data itself. Considering the highly modular nature of CNS data sets with specified layers of genes, proteins, metabolites, capsule networks can analyze the changes in the functional organization and interplay of these layers upon the diseases.

Another critical issue in the application of AI/ML models into CNS drug discovery is the integration of different data types, including genotypic data from patients, multiomics data from drug treatments, and chemical data from bioactivity and toxicity assays. Considering the availability of various databases that include biological, structural, and chemical information, how to integrate these data to generate AI/ML models becomes a critical question in CNS drug discovery applications. Multitask learning, learning of different tasks jointly, can be suitable for these types of applications. Multitask NNs are capable of integrating data from many distinct sources. For example, a multitask architecture can predict the effects of a drug and its BBB permeability at the same time by learning from multiomics data sets, physicochemical properties, HTS, and bioactivity assays.

In recent years, we have seen the emergence of novel neuroimaging techniques such as pharmacological functional magnetic resonance imaging (pharmacoMRI) and pharmacologically induced functional ultrasound (pharmaco‐fUS), which provide in vivo functional data of specific effects of drugs on the brain. Although pharmacoMRI continues to play a useful role in neuropharmacology studies as a well‐established technique,^458^, ^459^, ^460^, ^461^ a variety of challenges (i.e., low sensitivity, the requirement for anesthesia, and blood oxygenation‐level dependent imaging) limit the preclinical use of it. A newer tool, pharmaco‐fUS enables brain activity imaging through the local monitoring of cerebral blood volume dynamics at an unprecedented spatiotemporal resolution without the bias of anesthesia.^462^, ^463^ Recent studies demonstrated fUS imaging's potential to characterize dynamic profiles of CNS drugs, including a drug combination of donepezil plus mefloquine for AD^464^ and atomoxetine for attention‐deficit/hyperactivity disorder.^465^ Moreover, Rabut et al.^466^ adapted ML to analyze the rich data content provided by fUS connectivity imaging. Their ML model identified the “fingerprint” of drug‐induced brain connectivity changes in awake mice for scopolamine, a major preclinical drug to model AD. As evident from the previous applications, AI/ML methods hold the promise of characterization of treatment effects from novel neuroimaging data sets and thereby improving our understanding of the mechanism of action of drugs in the brain. Getting drugs across the BBB is an essential step to developing successful therapies to treat CNS disorders. However, it is often overlooked that BBB is not only a physical barrier for drug delivery to the CNS but also a complex, dynamic interface that might be affected by diseases. CNS disorders may result in dysfunction of BBB, such as its disruption or dysfunctions related to BBB transporters. To date, AI/ML‐based predictive algorithms have assumed that BBB is a static entity by neglecting the effects of CNS pathologies on it. Therefore, a prediction model for BBB penetrance that trained on data from non‐CNS diseases may not work for a CNS disease. To develop better prediction models for BBB permeability, we need to take into account disease‐related changes in the barrier. This also provides many unique opportunities for developing disease‐specific AI/ML tools in CNS drug discovery.

It is important to highlight that CNS drug discovery has a nondeterministic nature, where the neurological targets involve different pathways and their biological consequences are not the sums of the single functions, most drugs have diverse activities through multiple biological targets, and drug response is dependent on a range of factors (i.e., patient's genetic profile and drug's membrane permeability). Moreover, physiologic events are highly context‐specific: A receptor interaction may take place in the liver but not in the brain. AI/ML systems often fail to pick up such context‐specific nonlinear relationships and many other unknown contributing factors. As a result of incomplete domain representation, partial predictability in CNS drug discovery is inevitable. For example, an AI/ML algorithm may predict drug targets that neuroscientists know will likely have significant side effects in the brain or generate unsynthesizable molecules. Here, we need the human refinement process and hypothesis‐driven approach^467^ to address many of these challenges to achieve better performance. Knowledge acquisition from the human experts to the AI systems can help the AI/ML system learn and thereby guarantee the best scientific results. In consequence, this mixture of machine and mind^468^ will improve decision making as an essential component of the CNS drug discovery process.

Although AI/ML algorithms have already revolutionized other fields, the adoption of them to drug discovery is still at an eraly stage. Initially, AI/ML algorithms have been developed and practically used for certain areas such as image recognition, gaming, and internet search. Inspired by the successful applications in other disciplines, scientists have applied AI/ML algorithms to pharmaceutical research. And yet, we do not have any AI/ML algorithm that is developed specifically for a drug discovery problem. But this means that there should be many opportunities to develop innovative and novel algorithms in the field of therapeutic discovery. In this way, AI/ML methods will play an increasingly important role in not just the field of general pharmaceutical research but also CNS drug discovery.

In conclusion, we extensively review the latest AI/ML‐assisted drug discovery applications for the therapy of CNS diseases. These applications have been overgrowing in the past couple of years, fueled by the unprecedented success of AI/ML‐based approaches in different fields of science and technology. We envision that in the future, AI/ML will play more and more critical roles in CNS drug discovery towards personalized medicine, especially in the following areas: (1) patient subtyping, (2) identification of key disease drivers, (3) prediction of cell type‐specific drug response, (4) autonomous design of novel drugs, and (5) disease‐specific BBB permeability testing. Today there are structural constraints in data and algorithms that are limiting the role of AI/ML. Nonetheless, in the long run, ongoing and emerging developments in AI/ML approaches to neuropharmacology will enable us to develop more effective drugs for CNS diseases.
